# Supplementary material for: Synthesis of Highly Potent Anti-Inflammatory Compounds (ROS Inhibitors) from Isonicotinic Acid
Source: Molecules. 2021 Feb 26;26(5):1272. doi: 10.3390/molecules26051272 (PMC7956706; doi:10.3390/molecules26051272)
Supplement: Supplementary file 1 [file molecules-26-01272-s001.pdf]

molecules

MDPI

Communication

Synthesis of Highly Potent Anti-Inflammatory Compounds (ROS Inhibitors) from Isonicotinic Acid

Sana Yaqoob [1], Nourina Nasim [1], Rahila Khanam [1], Yan Wang [1], Almas Jabeen [2], Urooj Qureshi [1], Zaheer Ul-Haq [1,2], Hesham R. El-Seedi [3,4,5,*], Zi-Hua Jiang [6] and Farooq-Ahmad Khan [1,*]

[1] Third World Center for Science and Technology, H.E.J. Research Institute of Chemistry, International Center for Chemical and Biological Sciences, University of Karachi, Karachi-75270, Pakistan; sana.yaqoob@iccs.edu (S.Y.); nourina@iccs.edu (N.N.); rahila.khanam@iccs.edu (R.K.); yan.wang@iccs.edu (Y.W.); urooj.qureshi@iccs.edu (U.Q.); zaheer.qasmi@iccs.edu (Z.U.-H.)

[2] Dr. Panjwani Center for Molecular Medicine and Drug Research, International Center for Chemical and Biological Sciences, University of Karachi, Karachi-75270, Pakistan; almas@iccs.edu

[3] International Research Center for Food Nutrition and Safety, Jiangsu University, Zhenjiang 212013, China

[4] Department of Molecular Biosciences, The Wenner-Gren Institute, Stockholm University, S-106 91 Stockholm, Sweden

[5] Department of Chemistry, Faculty of Science, Menoufia University, Menoufia 32511, Egypt

[6] Department of Chemistry, Lakehead University, 955 Oliver Road, Thunder Bay, Ontario P7B 5E1, Canada; zjiang@lakeheadu.ca

* Correspondence: farooq.khan@iccs.edu (F.-A.K.); hesham.elseedi@su.se (H.R.E.-S.); Tel: +92-21-111-222-292 (F.-A.K.); +46-700-434-343 (H.R.E.-S.)

Citation: Yaqoob, S.; Nasim, N.; Khanam, R.; Wang, Y.; Jabeen, A.; Qureshi, U.; Ul-Haq, Z.; El-Seedi, H.R.; Jiang, Z.-H.; Khan, F.-A.; et al. Synthesis of Highly Potent Anti-Inflammatory Compounds (ROS Inhibitors). *Molecules* **2021**, *26*, 1272. https://doi.org/10.3390/molecules26051272

Academic Editor: Bogumil E. Brycki

Received: 13 January 2021

Accepted: 23 February 2021

Published: 26 February 2021

Publisher's Note: MDPI stays neutral with regard to jurisdictional claims in published maps and institutional affiliations.

Copyright: © 2021 by the authors. Licensee MDPI, Basel, Switzerland. This article is an open access article distributed under the terms and conditions of the Creative Commons Attribution (CC BY) license (http://creativecommons.org/licenses/by/4.0/).

1. Protocol for Anti-Inflammatory Assay

Oxidative burst method, which employs luminol-enhanced chemiluminescence technique, was used to evaluate the anti-inflammatory activity of the all the synthesized compounds [1]. Diluted human blood (25 µL) in buffer HBSS++ (Helfand Balanced Salt Solution purchased from Sigma-Aldrich®, St. Louis, USA), was treated with the compounds (concentrations: 1, 10 and 100 µg/mL; volume: 25 µL) each in triplicate. Control wells contained cells and HBSS^{++} in the absence of compounds. The compounds were incubated with blood at 37 ºC for 15 min in 96 well half area plate in the thermostatic luminometric chamber [Labsystems®, Finland]. 25 µL of serum opsonized zymosan (SOZ) obtained from Fluka® Switzerland, and 25 µL of luminol purchased from Research Organics®, USA were added, except into blank wells (containing only HBSS++ only). Relative light units (RLU) were used to count the level of reactive oxygen species (ROS) generation. Standard used for this assay was Ibuprofen with IC_{50} = 54.2 ± 9.2 µM

2. Docking Methodology for Anti-Inflammatory Activity

Molecular docking studies were performed to decipher cyclooxygenase-2 (PDB:4PH9) anti-inflammatory inhibitory mechanism by isonicotinate derivatives. The co-crystalized structure has a resolution of 1.81Å, cognate with standard anti-inflammatory drug Ibuprofen (IBP) [2]. The protein was prepared, and subjected to energetically minimize by Amber10 forcefield. Chemical architecture of isonicotinate series were constructed by Molecular-Operating-Environment (MOE) v. 2019.01 builder module, and minimized by MMFF94x forcefield. MOE was first utilized to benchmark the software reproducibility, by re-docking IBP molecule into the respective pocket. Afterwards, to investigate the binding modes of each active molecule of the series, Induce-fit docking was performed. The stability of molecules was confirmed by examining hydrophobic interactions and hydrogen bonding of molecules with the corresponding target via MOE-Protein Ligand Interaction Fingerprint (PLIF) Program. Other molecular modelling software used throughout this study was CHIMERA 1.14 [3]. Benchmarking of the docking software were performed by redocking Ibuprofen in the active pocket. The algorithms with least

square deviation between the initial and after dock pose i.e., 0.21Å were came out as, Triangle Matcher as placement method and London dG as scoring function with induce fit docking protocol implemented by MOE. Following that, isonicotinic acid derivatives were dock into the similar pocket as ibuprofen does.

3. NMR Spectra of The Active Compounds

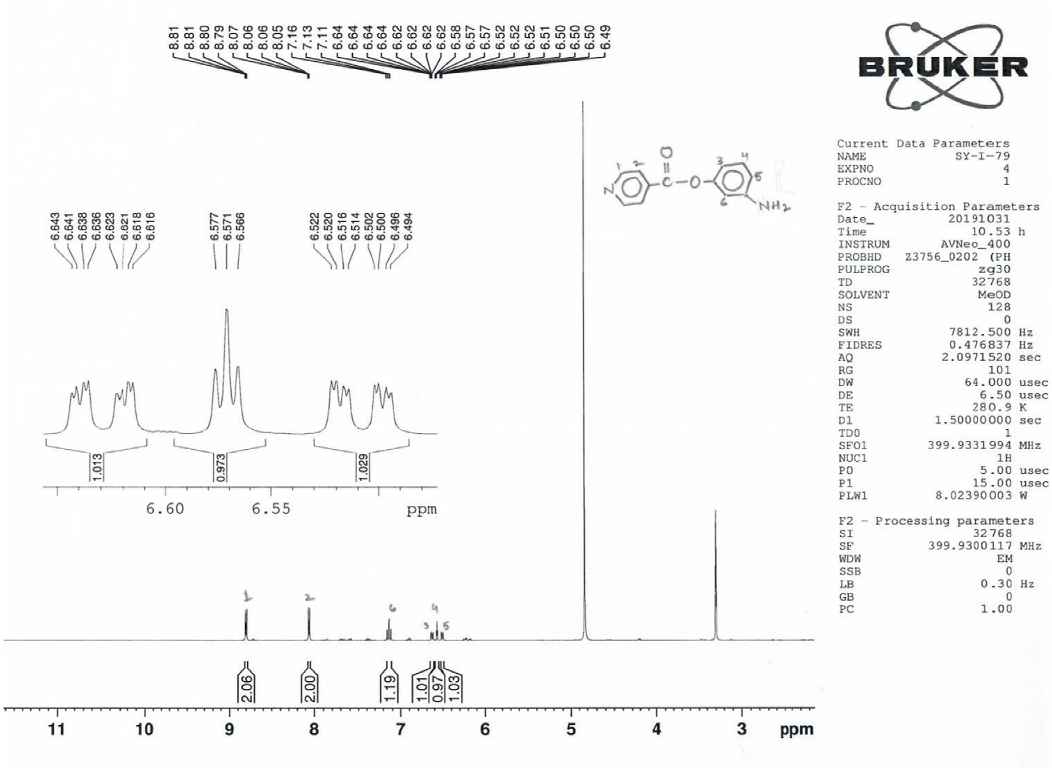

Figure 1. ^{1}H-NMR spectrum of *N*-(3-Aminophenyl) isonicotinamide (**5**).

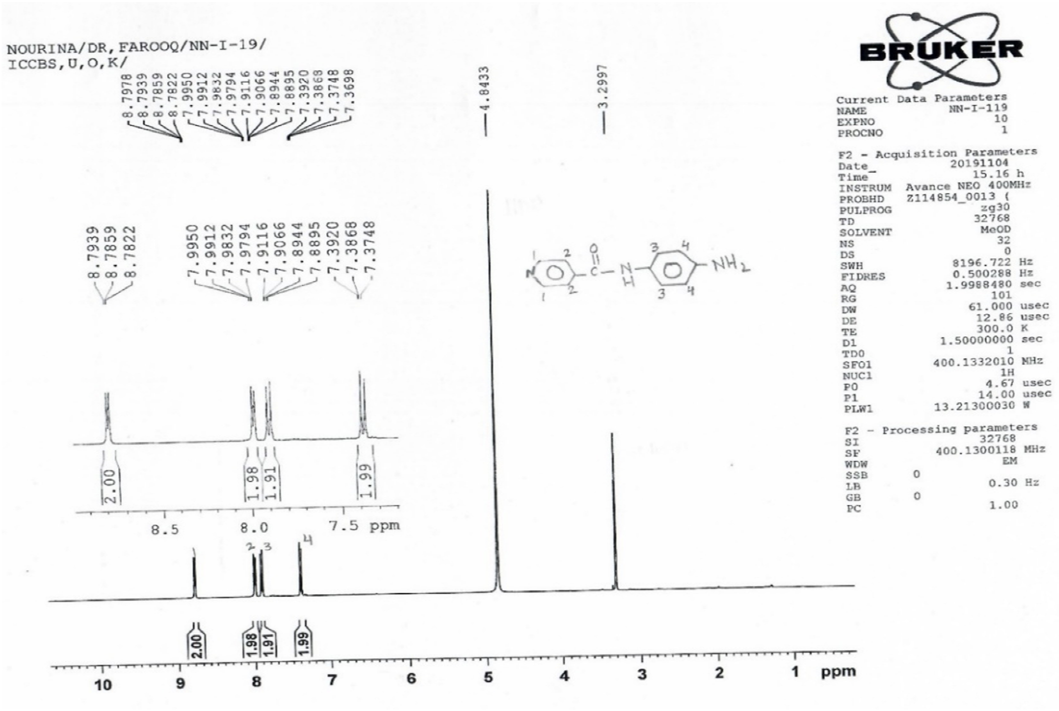

Figure 2. ^{1}H-NMR spectrum of *N*-(4-Aminophenyl) isonicotinamide (**6**).

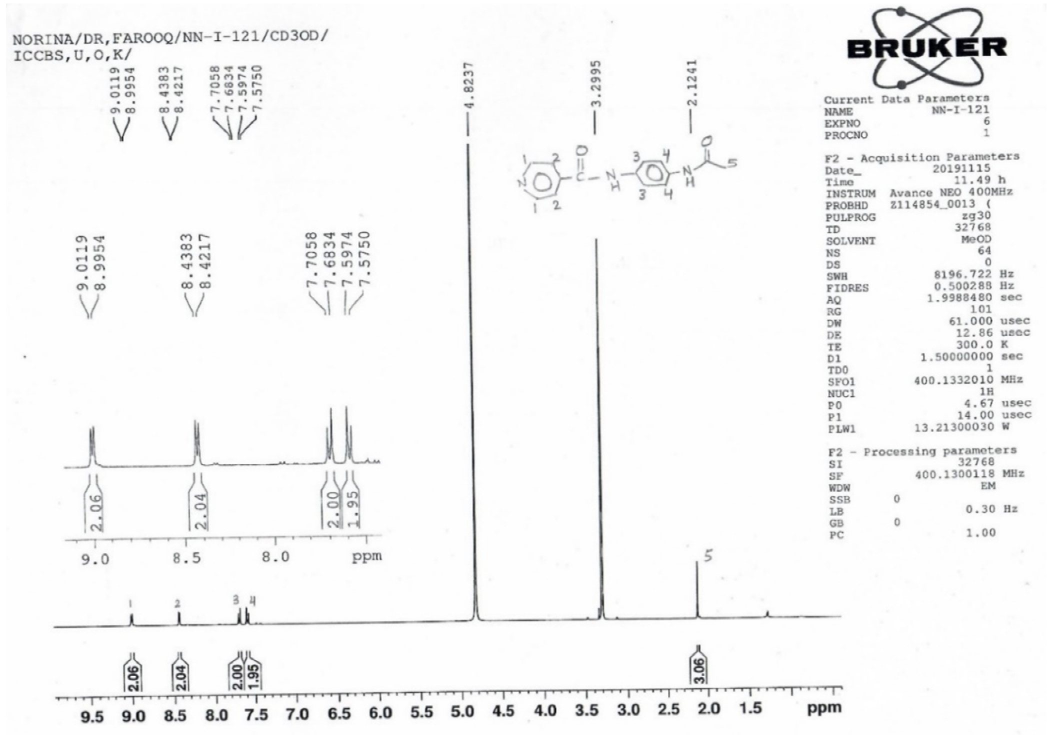

Figure 3. ^{1}H-NMR spectrum of *N*-(4-Acetamidophenyl) isonicotinamide (**12a**).

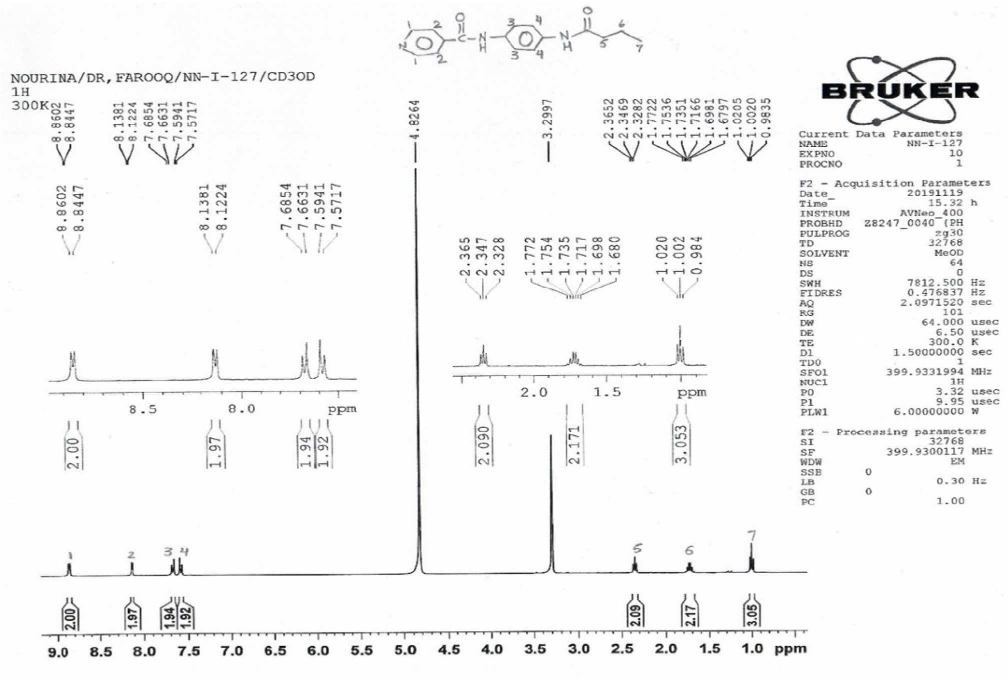

Figure 4. ^{1}H-NMR spectrum of *N*-(4-Butyramidophenyl) isonicotinamide (**12b**).

References

1. Helfand, S.L.; Werkmeister, J.; Roder, J.C. Chemiluminescence response of human natural killer cells: I. the relationship between target cell binding, chemiluminescence, and cytolysis. J. Exp. Med. 1982, 156, 492–505, doi:10.1084/jem.156.2.492.
2. Orlando, B.J.; Lucido, M.J.; Malkowski, M.G. The structure of ibuprofen bound to cyclooxygenase-2. J. Struct. Biol. 2015, 189, 62–66, doi:10.1016/j.jsb.2014.11.005.
3. Pettersen, E.F.; Goddard, T.D.; Huang, C.C.; Couch, G.S.; Greenblatt, D.M.; Meng, E.C.; Ferrin, T.E. UCSF chimera - A visualization system for exploratory research and analysis. J. Comput. Chem. 2004, 25, 1605–1612, doi:10.1002/jcc.20084.
